# Comparison of smartphone-based retinal imaging systems for diabetic retinopathy detection using deep learning

**DOI:** 10.1186/s12859-020-03587-2

**Published:** 2020-07-06

**Authors:** Mahmut Karakaya, Recep E. Hacisoftaoglu

**Affiliations:** Dept. of Computer Science, University of Central Arkansas, 201 Donaghey Ave, Conway, AR, 72035 USA

**Keywords:** Retinal imaging, Diabetic retinopathy, Deep learning, iExaminer, D-Eye, Peek retina, iNview

## Abstract

**Background:**

Diabetic retinopathy (DR), the most common cause of vision loss, is caused by damage to the small blood vessels in the retina. If untreated, it may result in varying degrees of vision loss and even blindness. Since DR is a silent disease that may cause no symptoms or only mild vision problems, annual eye exams are crucial for early detection to improve chances of effective treatment where fundus cameras are used to capture retinal image. However, fundus cameras are too big and heavy to be transported easily and too costly to be purchased by every health clinic, so fundus cameras are an inconvenient tool for widespread screening. Recent technological developments have enabled to use of smartphones in designing small-sized, low-power, and affordable retinal imaging systems to perform DR screening and automated DR detection using image processing methods. In this paper, we investigate the smartphone-based portable retinal imaging systems available on the market and compare their image quality and the automatic DR detection accuracy using a deep learning framework.

**Results:**

Based on the results, iNview retinal imaging system has the largest field of view and better image quality compared with iExaminer, D-Eye, and Peek Retina systems. The overall classification accuracy of smartphone-based systems are sorted as 61%, 62%, 69%, and 75% for iExaminer, D-Eye, Peek Retina, and iNview images, respectively. We observed that the network DR detection performance decreases as the field of view of the smartphone-based retinal systems get smaller where iNview is the largest and iExaminer is the smallest.

**Conclusions:**

The smartphone-based retina imaging systems can be used as an alternative to the direct ophthalmoscope. However, the field of view of the smartphone-based retina imaging systems plays an important role in determining the automatic DR detection accuracy.

## Background

The World Health Organization estimates that 347 million people have diabetes worldwide and the number will increase to 552 million by the year 2030. In the United States, more than 9 percent of Americans (29 million) have the disease and 8 million of those are undiagnosed. A diabetic person is at high risk of eye disease including diabetic retinopathy, diabetic macular edema, cataract, and glaucoma. The most common cause of vision loss is the diabetic retinopathy (DR) that is caused by bleeding of the small blood vessels in the retina. If the these bloody retina veins are untreated, it may cause to varying degrees of vision loss and even blindness. In the US, more than 4.4 million people aged 40 and older had DR problem at different stages. Due to the its silent nature, DR may cause no symptoms or only mild vision problems [1]. Since early detection could improve the chances of effective treatment to prevent blindness, doctors suggest the regular annual eye exams for diabetic patients. With early diagnosis and accurate evaluation of DR severity, it is possible to coordinate diabetic eye care and prompt appropriate treatment for prevention of blindness and visual loss [2]. However, current studies shows that access to such medical care in developed countries ranges between 60% and 90%, with significantly lower rates in developing countries [3]. Patients without eye care access do not benefit from the potential of early detection and getting effective treatment in a timely manner. Among rural and minority populations, there exist a significant disparity in early diagnosis and access to eye care.

In the retina examination, doctors use special optical devices such as an ophthalmoscope, 20D lens, and fundus camera. Due to the easier storage, better image quality, and faster electronic transfer, fundus cameras are widely used to detect eye diseases with their digital imaging features. However, retinal imaging with a fundus camera is a time-consuming and manual process. An expertise is required to capture a retinal image and an expert evaluation report may take a few days to submit. Since fundus cameras are also too big and heavy to be transported easily and too costly to be purchased by every health clinic, fundus cameras are inconvenient tools for widespread screening. In addition, it is very hard to find the equipment and expertise in rural areas that have a high diabetes rate [4]. The need of infrastructure for DR screening will become even more insufficient as the number of diabetic people increases. Therefore, there is a growing demand for small, portable, and inexpensive retinal imaging systems to perform DR screening.

Recent technological developments have enabled use of smartphones in designing small-sized, low-power, and affordable biomedical imaging devices. These systems are capable of imaging, onboard processing, and wireless communication. Therefore, smartphone-based systems are very popular in several applications ranging from health care to entertainment since they make existing systems small and portable. Since fundus cameras are large-size, heavy-weight, and high-price devices, they are good candidate to be transformed into a portable device to perform fast DR screening. Developing new portable retinal imaging systems using smartphones is an emerging research and technology area that attracts several universities and companies.

**Fig. 1:**
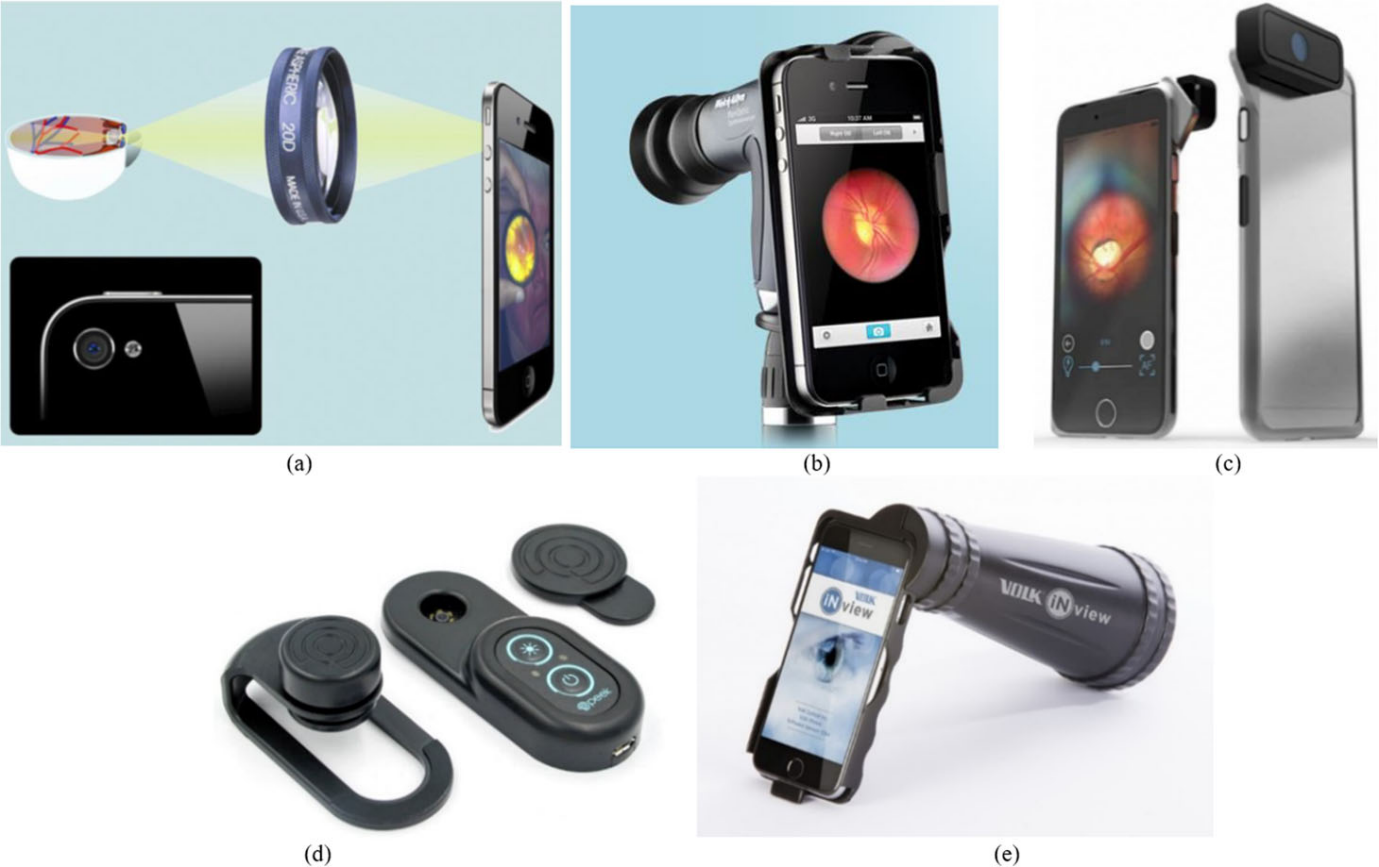
Smartphone-based retinal imaging systems available in the market, **a** Fundus photography method used by Harvard Medical School **b** iExaminer developed by Welch Allyn, **c** D-Eye, **d** Peek Retina, and **e** iNview by Volk

In the literature, using 20D Lens with a smartphone is one of the early designs to record video on human and rabbit eyes [5] as shown in Fig. 1a. This early design used the Filmic Pro mobile application to adjust focus, exposure, and light intensity manually. This allows having better quality fundus images in clinic settings for both under anesthesia and awake conditions. Another very simple smartphone-based design is the iExaminer system of Welch Allyn [6]. It is developed by attaching a smartphone to a PanOptic ophthalmoscope as shown in Fig. 1b. These systems require attaching the smartphone to an existing medical device. Recently, new smartphone-based retinal imaging systems are released to the market including D-Eye, Peek Retina, and iNview. These smartphone-based retinal imaging systems are bundled with associated secure HIPPA-compliant mobile application for image capture and transmission. In addition, iExaminer, D-Eye, and iNview have the Food and Drug Administration (FDA) approval for their devices. However, Peek Retina is currently waiting for the approval from FDA.

Russo et al. [7] developed the D-Eye, a small, portable, and inexpensive retinal imaging system to capture retina images as an attachment to a smartphone. Using the cross-polarization technique to reduce corneal reflections and integrated with smartphone’s autofocus feature to prevent patient’s refractive error, D-Eye allows retinal eye screening even for undilated eyes. To illuminate the retina, D-Eye reflects the smartphone flashlight as shown in Fig. 1c. Peek Retina imaging system [8] includes its own light to illuminate the retina and the image is captured by a smartphone as shown in Fig. 1d. To capture wide-angle retinal images, Volk Optical developed iNview smartphone-based retinal imaging system [9] as shown in Fig. 1e. It illuminates the retina by reflecting the smartphone flashlight and does not require pupil dilation to acquire 50 degrees of retinal view to visualize the entire posterior pole in a single image. All these smartphone-based imaging systems are capable of capturing retina images, but none of these smartphone-based systems offer a way to analyze and evaluate eye disease by using image processing techniques.

In addition, there exist several telemedicine solutions for retinal image analysis for diabetic retinopathy screening [10]. These solutions in literature need manual grading. However, they have to be fully automated to accelerate the diagnosis of retinal diseases using predictive models especially for patients in rural areas. Researchers presented automatic retinal image analysis (ARIA) tools including iGradingM [11], The TRIAD Network [12, 13], IDx-DR [14], RetmarkerDR [15], and Retianalyze [16]. However, these methods are semi-automated and require some expert control to decide the existence of the retinal disorder. This is the main obstacle to apply them automatically for large datasets.

Kaggle’s competition is one of attempts to attract researchers to present solutions for diabetic retinopathy detection providing the EyePACS retinal images. EyePACS contains 78685 retinal images to assess the DR existence and severity recommendation in different studies. This competition gives opportunity to researchers to design their deep learning networks and try them with a publicly available dataset. It also accelerates the research findings for automatic DR detection and usage of the deep networks for classification based medical research with higher detection performance. Graham is the winner achieving the accuracy score of 0.84957. He first preprocessed the retina images to remove the illumination difference and used a convolutional neural network, SparseConvNet and random forest for classification by augmenting the retina images to increase number of images in training set [17].

With the improvement of computational power and advances in neural network, deep learning algorithms, especially Convolutional Neural Networks (CNNs), have been widely used in different applications including retinal imaging. Rajalakshmi et al. [18] proposed using Remidio Fundus on Phone (FOP) device to capture high-quality retina images compared with the traditional fundus devices. The FOP is a high quality portable fundus camera that is capable of capturing 4 field retinal color photography covering macula, disc and nasal to the optic disc, superior-temporal and inferior-temporal quadrants. After data capturing, retina images are graded with EyeArt [19], a deep learning method to determine the existence of the diabetic retinopathy. It is a cloud-based retinal image assessment tool to grade DR development using deep learning methods that were trained with EyePACS dataset [20]. The EyeArt system also offers image processing and machine learning techniques such as image gradability, image enhancement, image restoration, interest region detection, and descriptor computation. Compared with manually graded results by two ophthalmologists, deep artificial neural network method shows high sensitivity and specificity for retina images captured by FOP device. FOP proves the concept of smartphone-based designs and shows the technological and economic feasibility of the portable retinal imaging systems. However, due to their fewer controllable parameters and inexpensive lenses, smartphone-based systems have a lower image quality compared to the fundus camera and FOP. Therefore, the existing algorithms could not be applied directly to the retinal images captured with smartphone-based retinal imaging systems because the quality of captured retina images plays an important role at the accuracy of the deep learning techniques.

In this paper, we first investigate the smartphone-based portable retinal imaging systems available on the market. We compare their image quality to determine if they are suitable for DR detection during a general health screening in urban areas, remote health clinics, nursing homes, pharmacies, schools, and even by individuals in their own homes. Then, we compare the DR detection accuracy of smartphone-based portable retinal imaging systems using a deep learning framework.

## Methods

In this section, we first present the details of smartphone-based portable retinal imaging systems available on the market to compare their features and image qualities. Second, we introduce the layout of the adopted deep learning architecture for DR detection.

### Smartphone-based portable retinal imaging systems

**Fig. 2:**
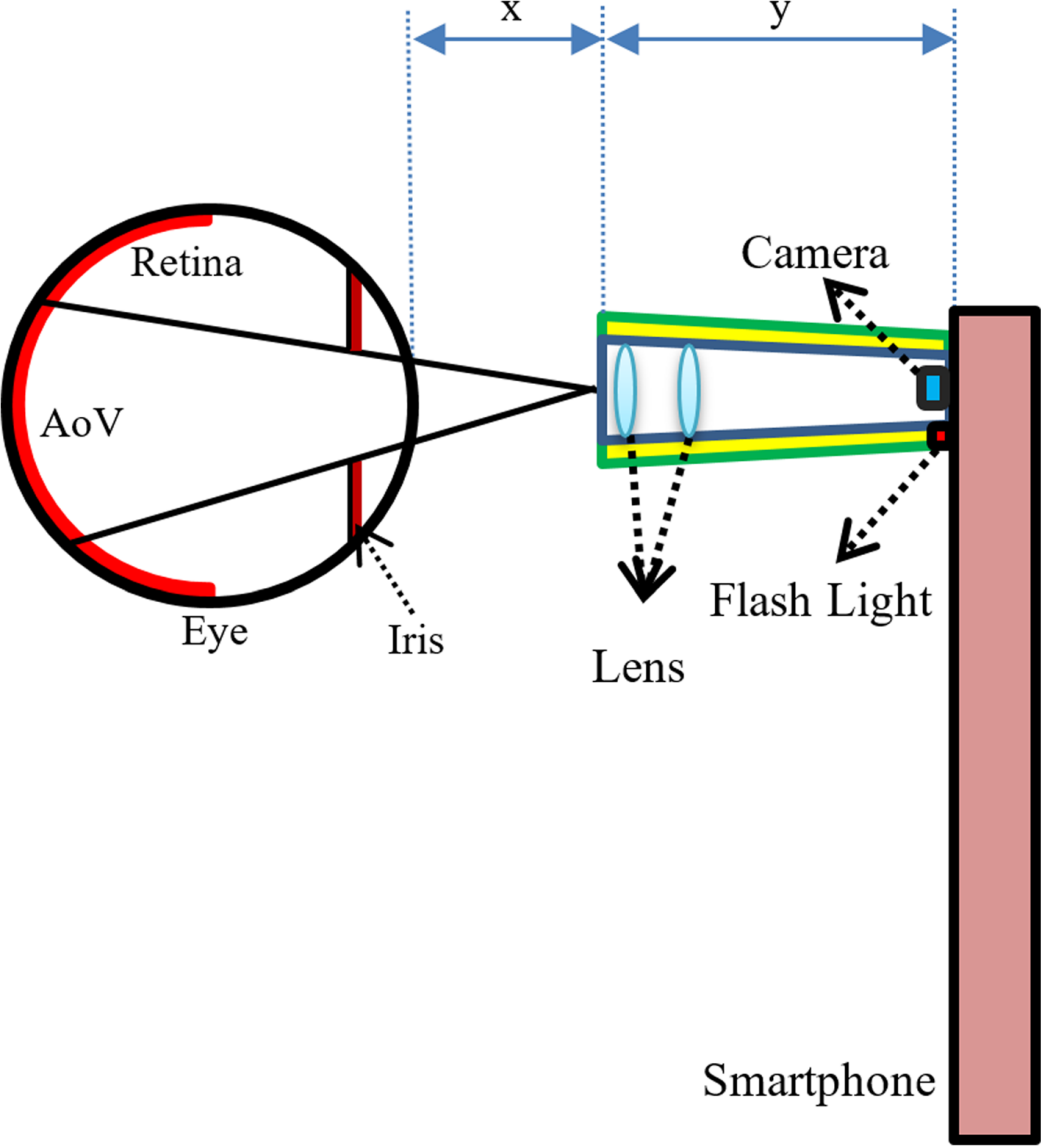
The general optical design of the smartphone-based retinal imaging devices

There are four smartphone-based retinal imaging systems available in the market including iExaminer, D-Eye, Peek Retina, and iNview. First, we attach each smartphone-based retinal imaging devices to the smartphone to capture retinal images. Since the retina is located at the back of the eye and light does not reflect back through the retina, it is not possible to take a picture of the retina directly without any external illumination. Therefore, these imaging systems attached to the smartphone needs to illuminate the dark retina by reflecting the smartphone flashlight on the retina or using its own light to brighten the retina. Figure 2 shows the general optical design of the smartphone-based retinal imaging devices. Based on their optical design, each smartphone-based portable retinal imaging system has different degrees in angles of retinal views (AoV) to capture retinal images. It suggests that this difference in angles determines the field of view of different imaging systems. For example, the distance of the device to eye, (i.e, x), the length of the device (i.e, y), number of lenses in the optic design, determines the angles of retinal views in different imaging systems.

**Fig. 3:**
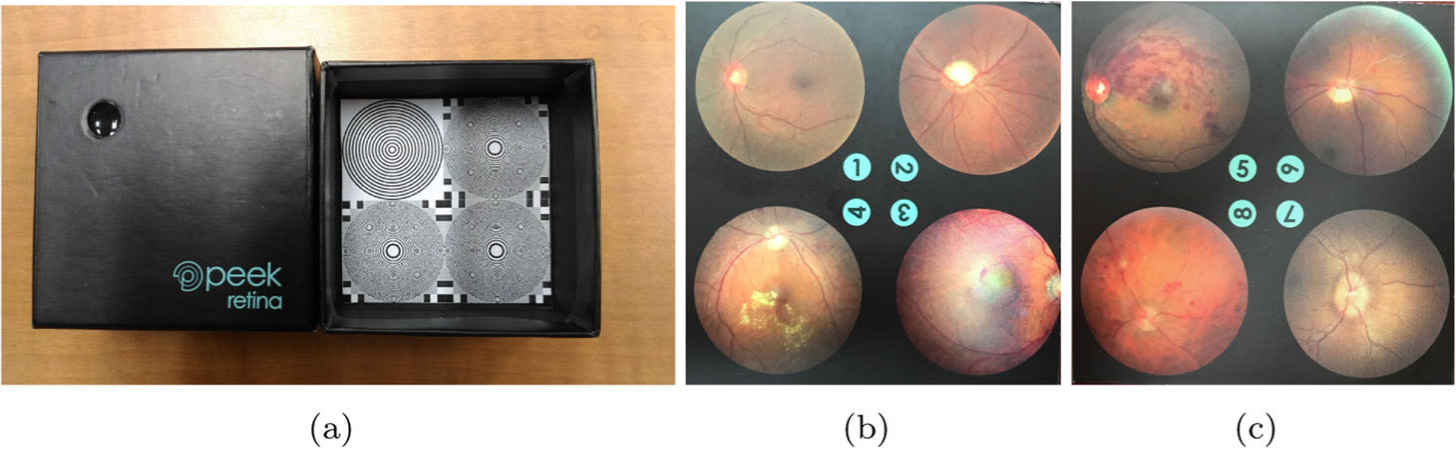
**a** Peek Retina Synthetic Eye Box for data collection and **b-c** Synthetic Retina Images

Even in a controlled environment, pupil dilation level of the human subject and the eye gaze between a human subject and the retinal imaging system changes easily. Since the visible portions of the retina will change, the retina images captured from a human subject changes over time for different smartphone-based retinal imaging systems. To fix the visible retina, a synthetic eye model can be used. For our experimental setup, we used the synthetic eye model box provided with Peek Retina smartphone-based retinal imaging system as shown in Fig. 3a. It replicates the eye structures including pupil, lens, and retina. The dimensions of the synthetic eye model are 53 mm x 53 mm x 22 mm in width, length, and depth. The opening on the box replicates the pupil of the eye. Under the pupil opening, there is a lens to illustrate the lens of the eye. Since the distance from the front surface of the cornea to the retina is approximately 24 mm on average, a printed test image is placed inside the box at the bottom that gives the same distance. As shown in Fig. 3b-c, the printed real retina images show related retina tissues including the optic nerve, macula, and blood vessel. There are eight different high-quality printed real retina images provided with Peek Retina system including (1) normal retina, (2) glaucoma, (3) age-related macular degeneration, (4) diabetic retinopathy - clinically significant macular oedema, (5) branch retinal vein occlusion, (6) diabetic retinopathy - ghost vessel, (7) papilloedema optic disc swelling, and (8) diabetic retinopathy - proliferative.

In the following subsections, we present the details about the publicly available smartphone-based portable retinal imaging systems and their features. Table 1 summarizes and compares the important features of smartphone-based retinal imaging systems publicly available in the market including their price, size, weight, compatible smartphones, illumination source, pupil dilation dependency, degree of retinal view, type of captured data with its own mobile application, image size, and the maximum number of images.

**Table 1:** Comparison of smartphone-based retinal imaging systems available in the market

	**iExaminer**	**D-Eye**	**Peek Retina**	**iNview**	**20 D Lens**
Compatible Smartphones	iPhone 6	iPhone 6/7	Universal	iPhone 5/6/6S	Universal
Illumination Source	Inside	Flashlight	Inside	Flashlight	Flashlight
Dilation dependency	Not Required	Required	Required	Not Required	Required
Degree of Retinal View	25	6-20	20-30	50	46
Working Distance (mm)	22	22	22	65	50
Size (mm)	70/220/162	68/135/7	25/75/35	180/76/180	50/50/10
Weight (g)	390	25	43	332	50
Price ($)	693	400	235	799	113
Type of captured data	Image	Video	Image/Video	Image	N/A
# of Image / Duration of Video	5 images	30 seconds	N/A	9 images	N/A
Size of Images/Video	320x280	640x480	640x480	640x480	N/A

#### 20D Lens

One of the early and simple design for smartphone-based retinal imaging is developed using 20D lens at Harvard Medical School and the Massachusetts Eye Hospital [5]. As shown in Fig. 1a, an operator holds the 20D lens directly in front of the eye and captures the retinal images with a smartphone. The flashlight of the smartphone or an external light source attached to the doctor’s head is used to illuminate the retina. After capturing images using any smartphone application, retinal images are extracted using the virtue of MovieTolmage and Video2Photo applications. In addition, light intensity, focusing, and exposure can be adjusted before data capture. In order to acquire the retinal images for optimal quality, not only the pupil dilation is suggested but also it requires a certain level of expertise to capture retinal images.

#### iExaminer

IExaminer is the another simple early design for fundus photography method using a smartphone [6]. It is developed by attaching a smartphone to a Welch Allyn PanOptic ophthalmoscope. PanOptic Ophthalmoscope has been used for several years by ophthalmologists to provide convenient eye exams and detect eye diseases, including diabetic retinopathy (DR). In the iExaminer system, the eyecup of the ophthalmoscope where the doctor looks is removed and the smartphone is attached using a special attachment to capture the retina images using the smartphone’s camera. The iExaminer system involves three main pieces, including a smartphone application, iExaminer adapter, and PanOptic Ophthalmoscope. However, tt is only compatible with the iPhone 4 and 6 since there is no smartphone adapter available in the market for other devices.

To capture retina images, iExaminer provides up to 25-degree field-of-view for dilated eyes and allows adjusting focus ranging from -20 to +20 diopter. It offers several apertures and filter options including small spot, large spot, microspot, slit aperture, red-free filter, cobalt blue filter, half-moon, and fixation aperture. Its optic design generates its own light, converges it to a point at the cornea, and diverges around the retina. This allows easy entry into small pupils and wide area illumination of the fundus. Therefore, it does not require pupil dilation for retinal imaging. The operator can also control the amount of illumination manually. It also has an eyecup at the patient side that helps stabilization for the view and occludes ambient light to prevent the interference from outside light. The smartphone application allows capturing up to five images from a patient. After capturing retina images, the iExaminer system can send them to the doctor via an e-mail without applying any image processing algorithms.

**Fig. 4:**
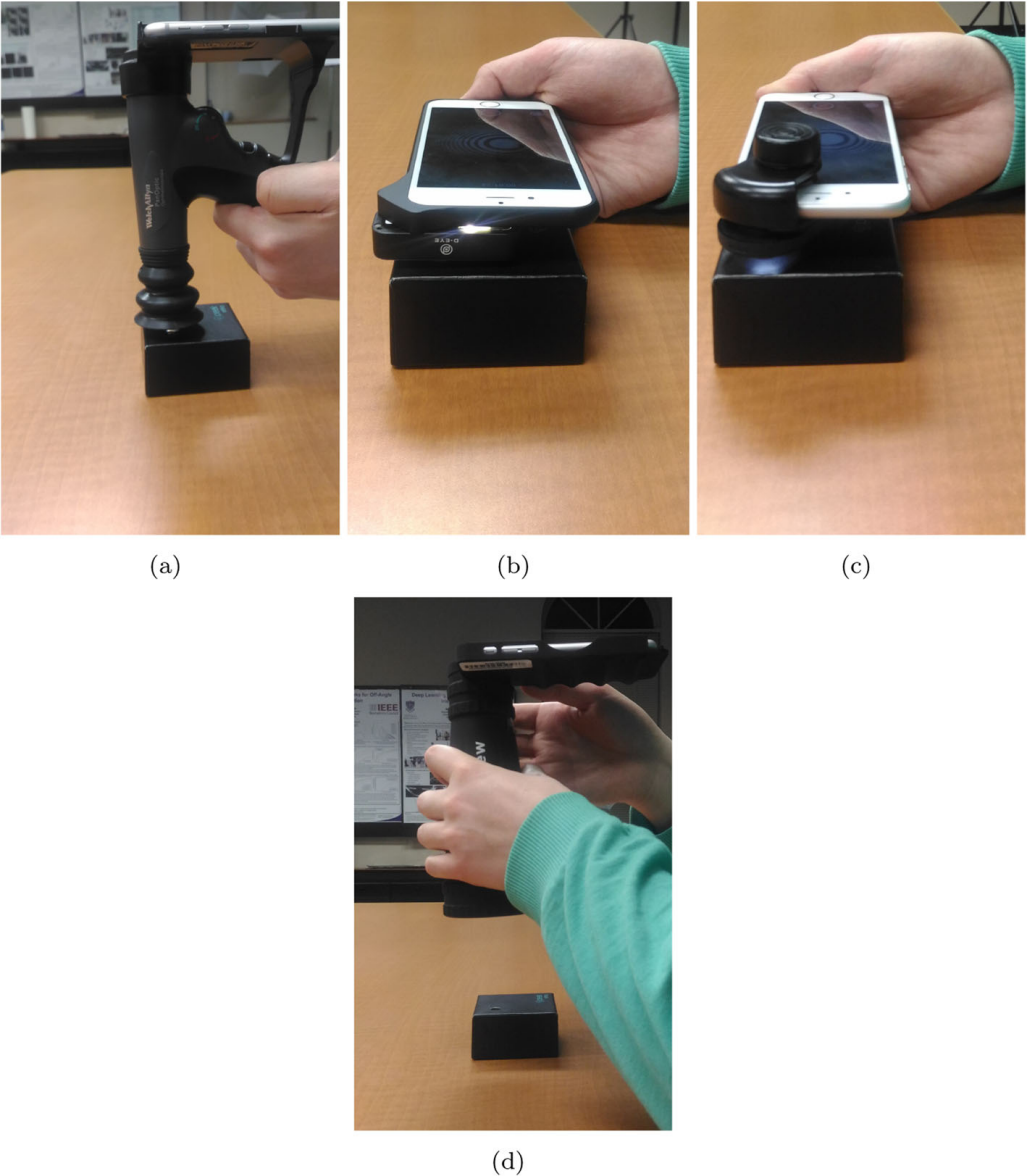
Experimental setup retinal imaging with **a** iExaminer, **b** D-Eye, **c** Peek Retina, and **d** Volk iNview

To capture images with iExaminer, we attached the phone to the adapter on the PanOptic Ophthalmoscope. Since iExaminer uses its own light, we disabled the flashlight of the phone and set the carousel settings for large light in the ophthalmoscope. Then, we positioned the iExaminer on the synthetic eye model box and touched the eyecup to the box as shown in Fig. 4a. In order to use the auto-focus property, we set the diopter to zero and waited for a few seconds before capturing the images.

#### D-Eye

D-Eye retinal imaging system [21] illuminates the retina by reflecting the smartphone flashlight to capture magnified retinal images up to 20 degrees in angle using its optics design and the smartphone camera. The D-Eye adapter is designed to attach it to various compatible iPhone models using compatible bumpers to magnetically attach. Since D-Eye reflects the flashlight of the smartphone to the retina using its optic design with mirrors and lenses, it does not require additional external power and light sources. Due to this design constraint, D-Eye is compatible with iPhone 5, 5s, 6, 6Plus, 6s, 6s Plus, and 7. D-Eye also provides an iOS application that gives the opportunity to both recording videos and shooting multiple images. Even if the system does not require pupil dilation to capture retina images, pupil dilation might be helpful where a larger field of view is needed such as analyzing retinal tissue structures and screening complete retina. For undilated eyes, D-Eye system can capture retina up to a 6-degree angle of view, while dilated eyes allow the 20-degree angle of view. To capture images with D-Eye, we position the D-Eye on the synthetic eye model box as close as possible as shown in Fig. 4b.

#### Peek retina

Peek Retina is another plug-in imaging system [8] that has its own adjustable light and power source to illuminate the retina. Users can change the amount of light to illuminate the retina by choosing one level out of three. Since it has its own light source, Peek Retina has a universal clip to attach any smartphone. It also has own Android application, named Peek Retina Camera that enables to capturing photos and recording videos. However, we can use any camera application with iPhone and Windows Phone models to capture images by adjusting manual settings in terms of autofocusing, clarity, and brightness.

Since Peek Retina system still waiting for approval from the Food and Drug Administration in the US, it can only be used for educational purposes not for medical purposes. However, there is a synthetic eye model box in its package for synthetic data capture. Therefore, we can only collect synthetic data provided in its package with eight different retina images that have different eye disease conditions from the normal eye to proliferative diabetic retinopathy. For data capture with Peek Retina, we positioned the Peek Retina adapter to align with the camera and tighten the knob to hold the device in the correct position as shown in Fig. 4c. Since Peek Retina has its own light source, we disabled the flashlight of the smartphone.

#### Volk iNview

The Volk iNview [9] smartphone-based retinal imaging system is developed by Volk Optical to capture wide-angle retinal images. It has three core components, including a mobile application, indirect ophthalmoscopy lens, and attachment adaptor for iPhone or iPod. This system captures the high-resolution retinal images using the camera and flashlight of the smartphone. The system does not require pupil dilation and it is able to capture 50 degrees of retinal view to visualize the entire posterior pole in a single image. In order to magnify the retina and to visualize it in a wider field of view, iNview Retinal Imager used 25D (diopter) lens as the primary imaging lens in its design. Its optical design generates a working distance of approximately 65mm. Due to the size of the 25D lens in the optical design, it has a large end tube size that requires to the operator to use the patient’s forehead as a steady reference to stabilize the device.

iNview provides the widest field of view compared to other smartphone-based retinal imaging systems but it is also the biggest and heaviest design (see Table 1). iNview is compatible with iPhone 5s, 6, 6s and iPod Touch. The free mobile application provides automatic retinal image capture and good auto-focus features. It also allows the user to encrypt the captured images using a password key for data security. This adds an extra layer of security to send the retinal images through an e-mail. Since iNview does not have its own light, we enabled the phone flashlight and positioned the iNview on the synthetic eye model box. In order to use the auto-focus property, we placed the iNview to about 150mm away from the box and make it closer to box to set the distance around 65mm (the best distance for data acquisition) as shown in Fig. 4d. Waiting for a few seconds before data capture helps the auto-focus feature to stabilize the image.

### Deep learning architecture for diabetic retinopathy detection

This section provides the layout of the utilized deep learning framework. For image classification, Convolution Neural Network (CNN) is one of the most popular deep learning frameworks so this work adopts the CNN based AlexNet [22] using the transfer learning. Due to its simple design and high classification accuracy compared with other CNNs, AlexNet is very popular in different research communities and used in several applications. Since 1.2 million high-resolution images are used to train AlexNet, it classifies images into 1000 different classes with a very low error rate.

AlexNet first uses five convolutional layers to extract low-level features. Then, it maps the final features to the set of the pre-determined number of classes using three fully connected layers and a softmax layer. In order to transfer the network knowledge, we use the general purpose features learned previously to retrain the softmax layer on a different dataset with different classes. In each layer, network architecture contains different layers such as convolutions, fully connected, activation functions, dropouts, and max-pooling. More complex features are extracted gradually in each convolution layer using the features from previous layers in the network. To introduce nonlinearity into the network, we use Rectified Linear Units (ReLU) as an activation function after each convolutional layer and fully-connected layer. Max pooling layers are used to keep the number of parameters required to be learned by outputting the maximum value of each square kernel and discarding the rest. This helps to reduce the redundancies among the features learned by the network. Dropout layers are introduced after the last two ReLU activations during training to force the network learning more robust features from training samples and avoid the overfitting problem. Using the output of the last fully connected layer, softmax classify images into different classes based on the highest probability.

Since AlexNet contains nearly 60 million parameters in its architecture, training it from scratch requires a very large amount of input images and computational power. When we do not have millions of labeled image, the best approach is to use transfer learning. In this paper, we removed the last three layers of the AlexNet (i.e., 1000-class fully connected, softmax, and classification). Then, we replaced them with new two-class fully connected layer, softmax, and classification layers for transfer learning. Finally, we retrained the new network with the retina images from EyePACS dataset using Stochastic gradient descent (SGD) algorithm with a minibatch size of 2 examples, the learning rate of 1*e*−5, and a momentum of 0.9. During the retraining, the network updates the parameters of each layer for every iteration with each training sample.

For all our experiments, we developed our programs and CNN frameworks using the MatConvNet and image processing toolboxes in MATLAB 2018. The experiments are performed on a DELL Alienware Aurora R8 workstation with 8 core Intel Core i7 9700K processor at 4.6GHz, NVIDIA GeForce RTX 2080 with 8GB GPU, and 32GB memory. Training time for transfer learning is around 117 sec for 686 images.

## Results and discussion

To compare the smartphone-based retinal imaging systems, we set up two sets of experiments using synthetic retina images and real fundus images. We first collected retina images and videos using iExaminer, D-Eye, Peek Retina, and iNview. To make the fair comparison between different retinal imaging systems, we captured retinal images with iPhone 6 using their compatible adapters and bumpers. In order to capture an image of the dark retina, we first needed to use a light source for illumination. For this purpose, we reflected the smartphone flashlight to the retina for D-Eye and iNview or use its own light source on iExaminer and Peek Retina. The smartphone’s camera was used to capture retina images and images were saved to smartphone’s memory.

### Retinal imaging with smartphone-based systems

In our first set of experiments, we captured images from the printed retina in the retina box using different smartphone-based retinal imaging systems. We placed the printed real retina images at the bottom of the Peek Retina synthetic eye model box as shown in Fig. 3. Then, we captured retina images from the optimum distance of each device in order to get the largest field of view as shown in Fig. 4. With this set of experiments, we compared the image quality and field of view of smartphone-based retinal imaging systems.

**Fig. 5:**
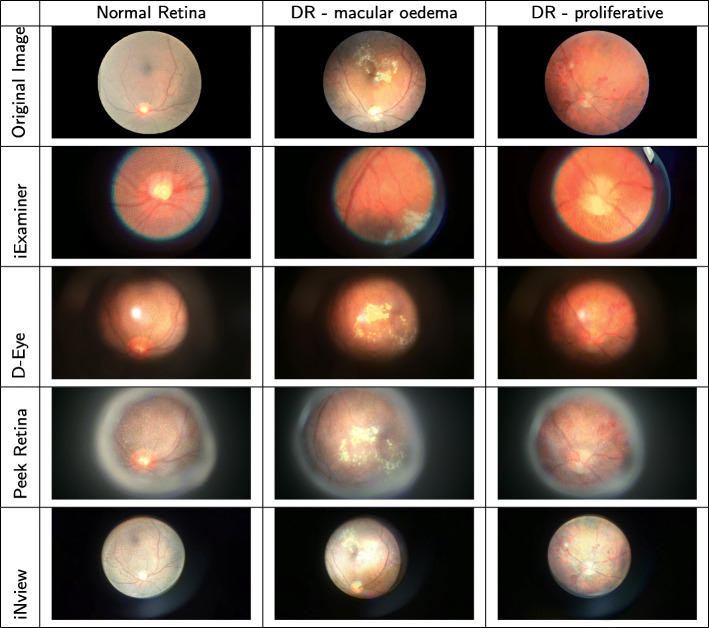
Retina images. (1st row) Original printed real retina images, and their captured versions using (2nd row) iExaminer, (3rd row) D-Eye, (4th row) Peek Retina, (5th row) iNview

Figure 5 shows the captured printed real retina images using iExaminer, D-Eye, Peek Retina, iNview. The original printed real retina images are shown in the first row. The image at the top left corner is a normal retina image where there is no abnormality is visible. The image at the middle of the top row belongs to a diabetic retinopathy patient where there is clinically significant macular edema. The image on the right of the top row belongs to a proliferative diabetic retinopathy patient where there are several visible abnormalities. The rest of the images on each row are captured using smartphone-based retinal imaging systems from retina box for each original printed real retina images at the first row. Images at the second row are captured using iExaminer. We observe that the image quality is good and the optic nerve and some central blood veins are visible. Since some macula edema can be seen, these images can be used for diabetic retinopathy detection. Images on the third row are captured using D-Eye where the illumination does not distribute evenly. However, we can still visualize the macular edema so they are helpful for diabetic retinopathy detection. Images on the fourth row of Fig. 5 shows the captured using Peek Retina. We observed that the field of view of Peek Retina is larger than iExaminer. The optic nerve and its surrounding vessels are visible, so these images can be used to detect diabetic retinopathy. The images at the last in Fig. 5 are captured using iNview. We observe that its field of view is the largest compared with other smartphone-based retinal imaging systems. The image quality is very good with evenly distributed illumination.

**Fig. 6:**
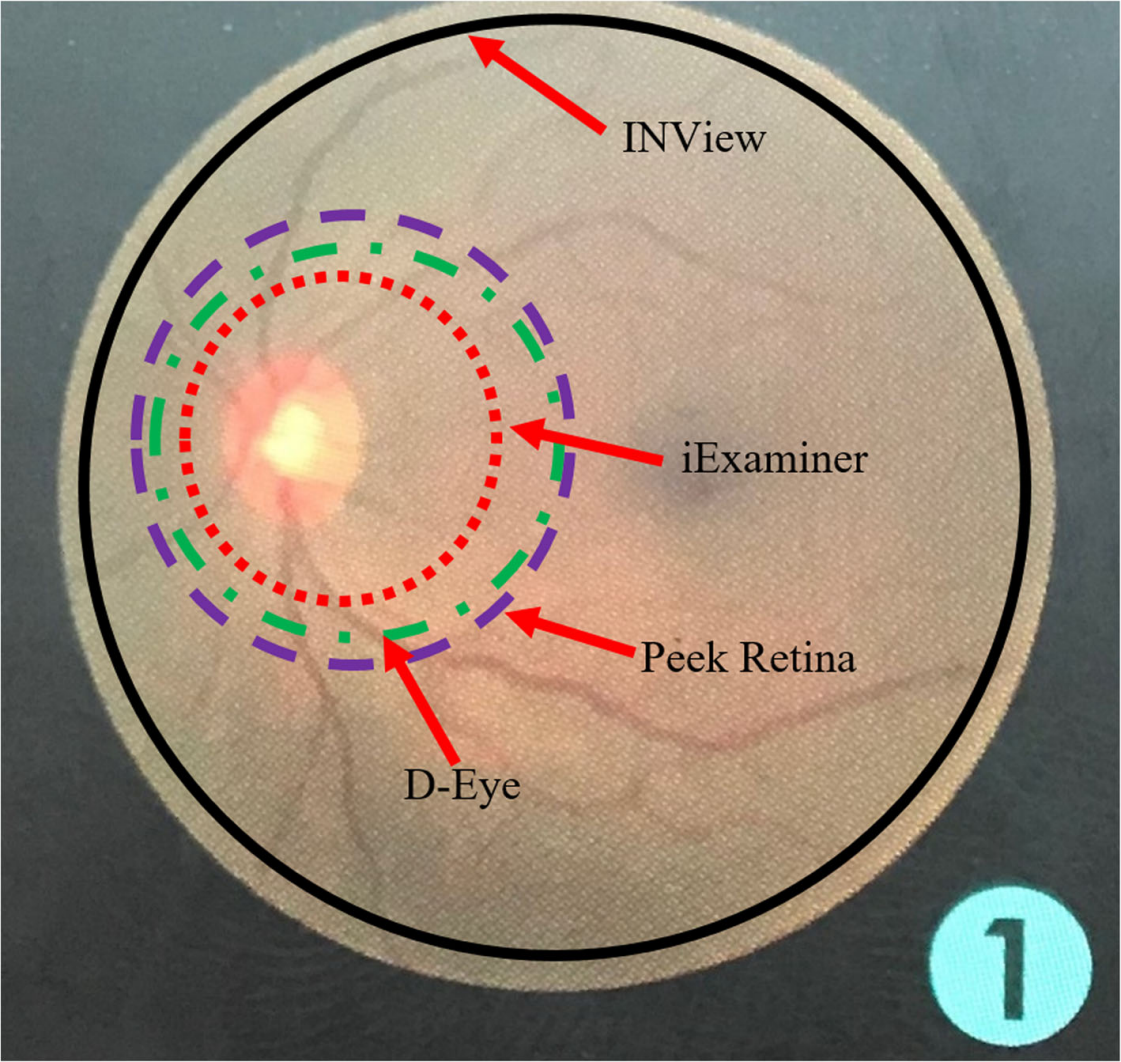
Comparison of the field of view of each smartphone-based retinal imaging system using printed normal retina image with field of view markers where the solid black line is for iNview, the green dashed-dotted line is for D-Eye, purple dashed line is for Peek Retina, the red dotted line is for iExaminer

To compare the field of view of the retinal imaging systems, Fig. 6 shows the captured printed retina images using iExaminer, D-Eye, Peek Retina, and iNview, respectively. Even if the printed retina image shows a larger portion of the retina, none of the smartphone-based retinal imaging systems can capture the entire printed retina pattern. Fig. 6 shows the printed image from a normal retina with the markers for the field of view of each imaging system. We observed that the radius of field of views for iExaminer, D-Eye, Peek Retina, and iNview are 32%, 40%, 45%, and 94% of the radius of printed fundus image, respectively. iNview has the largest field of view compared with others where its field of view is marked with the solid black line. D-Eye has the second largest field of view that is marked with a green dashed-dotted line. Peek Retina has almost the same size field of view with D-Eye marked with a purple dashed line. However, their field of view is almost half of the iNview. The smallest field of view belongs to iExaminer where it is only possible to capture fovea and its surroundings that is marked with a red dotted line.

### Diabetic retinopathy detection with deep learning

**Fig. 7:**
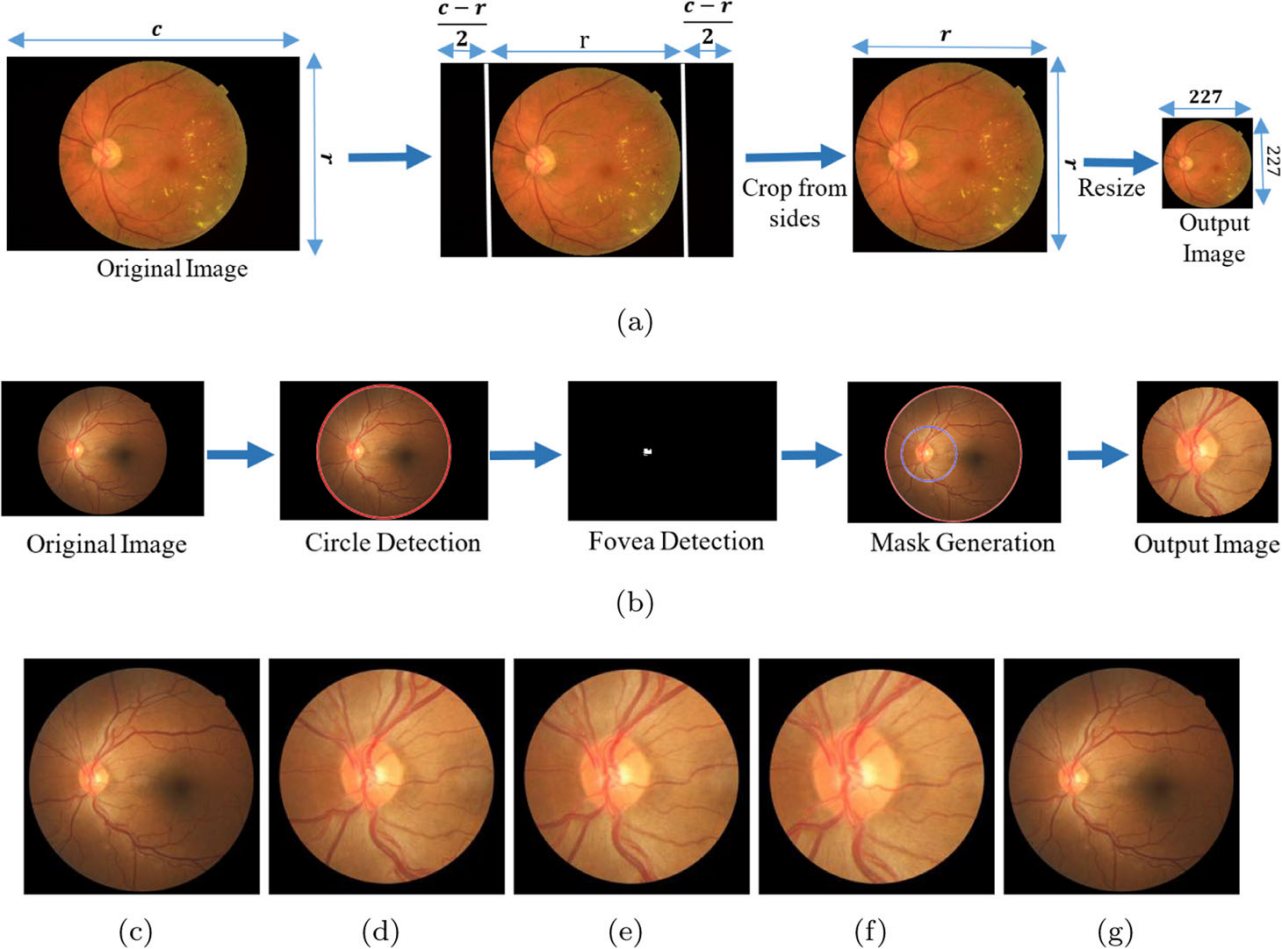
**a** Flow chart of original retina conversion images for AlexNet, **b** Flow chart of synthetic retina image generation for smartphone-based retinal imaging systems. Example of images, **c** Original image from EyePACS dataset and generated synthetic images for **d** iExaminer, **e** D-Eye, **f** Peek Retina, **g** iNview

In our second set of experiments, we design several experiments for each smartphone-based retinal imaging device to analyze the DR detection performance of the deep learning framework for smartphone-based systems and compare them with the accuracy of original retina images. The original retina images in the dataset are originally color images and their resolution vary since they captured by different fundus cameras. However, AlexNet Deep Learning framework requires the inputs to have 227x227x3 pixels as color images. Therefore, we first cropped pixels from right and left sides of each original image to make it square shape. Then, we down-sampled the cropped square images into 227x227x3 as shown in Fig. 7a.

In order to train and test the deep learning network, we need to feed the CNN frameworks with retina images captured by different smartphone-based devices. However, there is no available real data captured by the smartphone-based retinal imaging device in the literature. Therefore, we generate retina images by simulating the field of view for each device using the retina images from EyePACS dataset as illustrated in Fig. 7b. We first segment the boundary of the original retina images in EyePACS and fit a circle to its boundary using circular Hough transform. Second, the optic nerve is detected using thresholding and morphological filters since the optic nerve is the brightest area in the retina. Then, we generate a circular mask of each smartphone-based device using the center of optic nerve as a circle center. The mask radius is calculated by multiplying the radius of the original image boundary and the percentage of the radius of field of views in Fig. 6. Finally, the original image is masked and cropped with respect to the mask center. Examples of synthetic retina images for iExaminer, D-Eye, Peek Retina, iNview are shown in Fig. 7c-g, respectively.

**Fig. 8:**
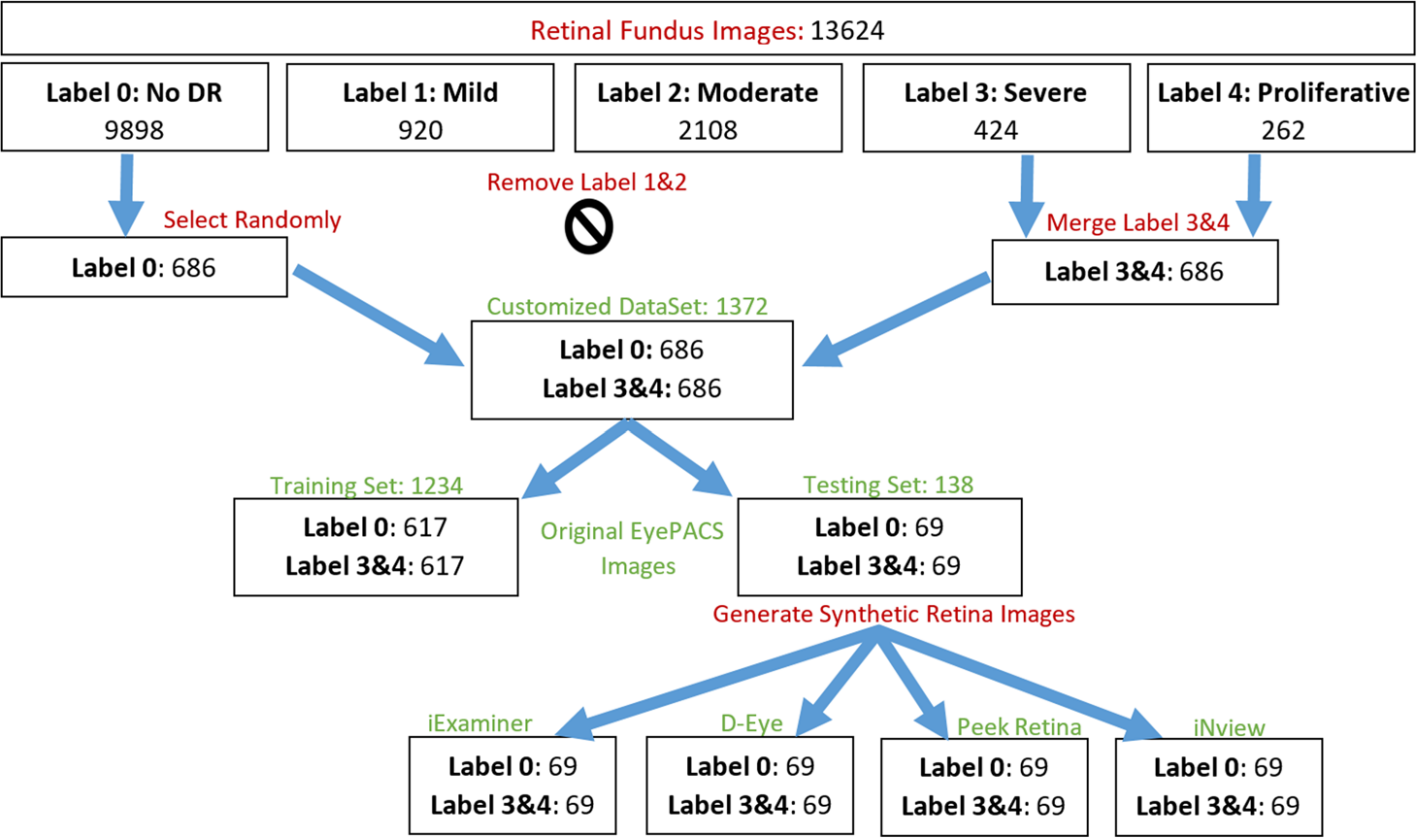
Generation of customized dataset of retinal fundus images and its label distribution

In the original image dataset, we have 13624 retina images from five different gradings (i.e., 0:No DR, 1:Mild, 2:Moderate, 3:Severe, and 4:Proliferative DR) where their distributions are 9898, 920, 2108, 424, and 262, respectively. In order to simplify the problem, we dropped the grade 1 and 2 and merged grade 3 and 4. Since no DR images are more than other labels in the dataset, we randomly selected 686 no DR images and removed the rest from the dataset in order to remove the data bias. As a result, we had 686 no DR and 686 DR images remaining in each subset as shown in Fig. 8. Then, we split our customized dataset with a ratio of 1/9 into two subsets where the testing subset has 1234 images and the training set has 138 images. We selected the bigger subset of original retina images as a training set and the second subset from each synthetic retina subsets are used as testing sets. After training the network with 1234 original retinal images, we tested the network with 138 retinal images from each datasets including original, iExaminer, D-Eye, Peek Retina, and iNview images. In total, we used 690 retinal images from five different datasets.

**Fig. 9:**
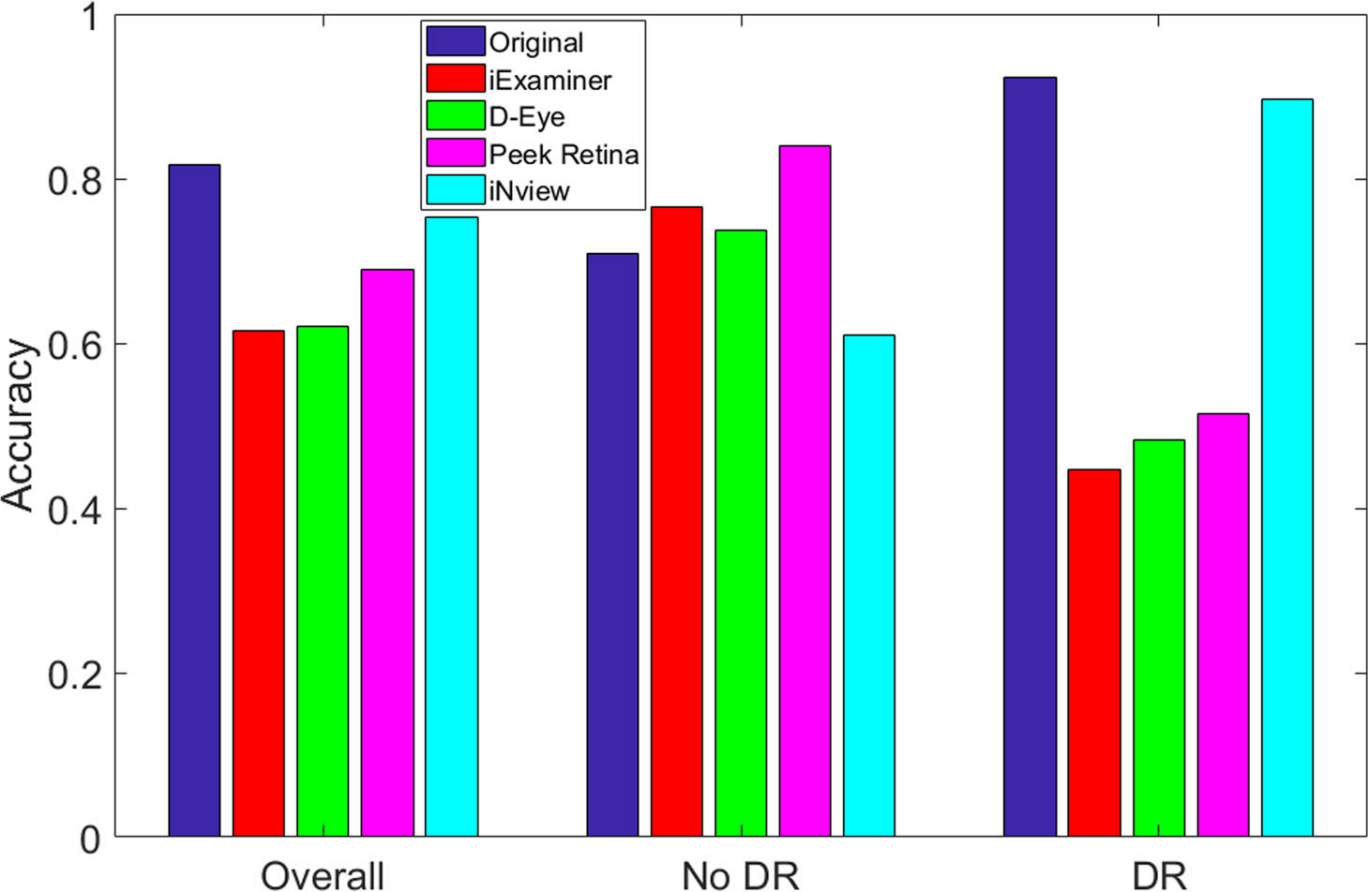
Classification accuracy of deep learning network for different testing images from original and synthetic smartphone-based retinal images

Table 2 compares the classification accuracy of the deep learning network for different testing images from original and synthetic smartphone-based retinal images. We repeated the test with different sub-sampling rates of test images and their mean and standard deviation values are given in Table 2. Since the network is trained with original images, it shows the highest overall accuracy (82% with a standard deviation of 0.0258) for testing with original images, as expected. The overall classification accuracies of smartphone-based systems are sorted as 61%, 62%, 69%, and 75% for iExaminer, D-Eye, Peek Retina, and iNview images, respectively. We observed that the overall network performance increases as the field of view of the smartphone-based retinal systems get bigger where their radius of printed fundus image are 32%, 40%, 45%, and 94% for iExaminer, D-Eye, Peek Retina, and iNview, respectively. We also observed that the accuracy of detecting healthy retina (No DR) is higher than retina with DR, especially for iExaminer, D-Eye, and Peek Retina images. In addition, the accuracy of both labels is close to each other for iNview images. The main reason is that iExaminer, D-Eye, and Peek Retina systems can capture smaller areas of the retina and mainly their images are focused on the optic disk and its surroundings. However, lesions generally appear on areas away from the optic disk. Since iNview captures a wider field of view, where it is more probable to include lesions, it detects DR better than other smartphone-based retinal imaging systems. For better visualization, we also show the overall, No DR, and DR classification accuracies in Fig. 9 for original, iExaminer, D-Eye, Peek Retina, and iNview images.

**Table 2:** Classification accuracy of deep learning network

	Overall	No DR	DR
Original	0.8164 ±0.0258	0.7094 ±0.0431	0.9234 ±0.0372
iExaminer	0.6144 ±0.0552	0.7661 ±0.0582	0.4464 ±0.0894
D-Eye	0.6211 ±0.0593	0.7371 ±0.0566	0.4827 ±0.0784
Peek Retina	0.6889 ±0.0353	0.8397 ±0.0451	0.5140 ±0.0539
iNview	0.7531 ±0.0311	0.6094 ±0.0491	0.8969 ±0.0394

Note that each result shows the mean and its standard deviation as mean ±std

**Fig. 10:**
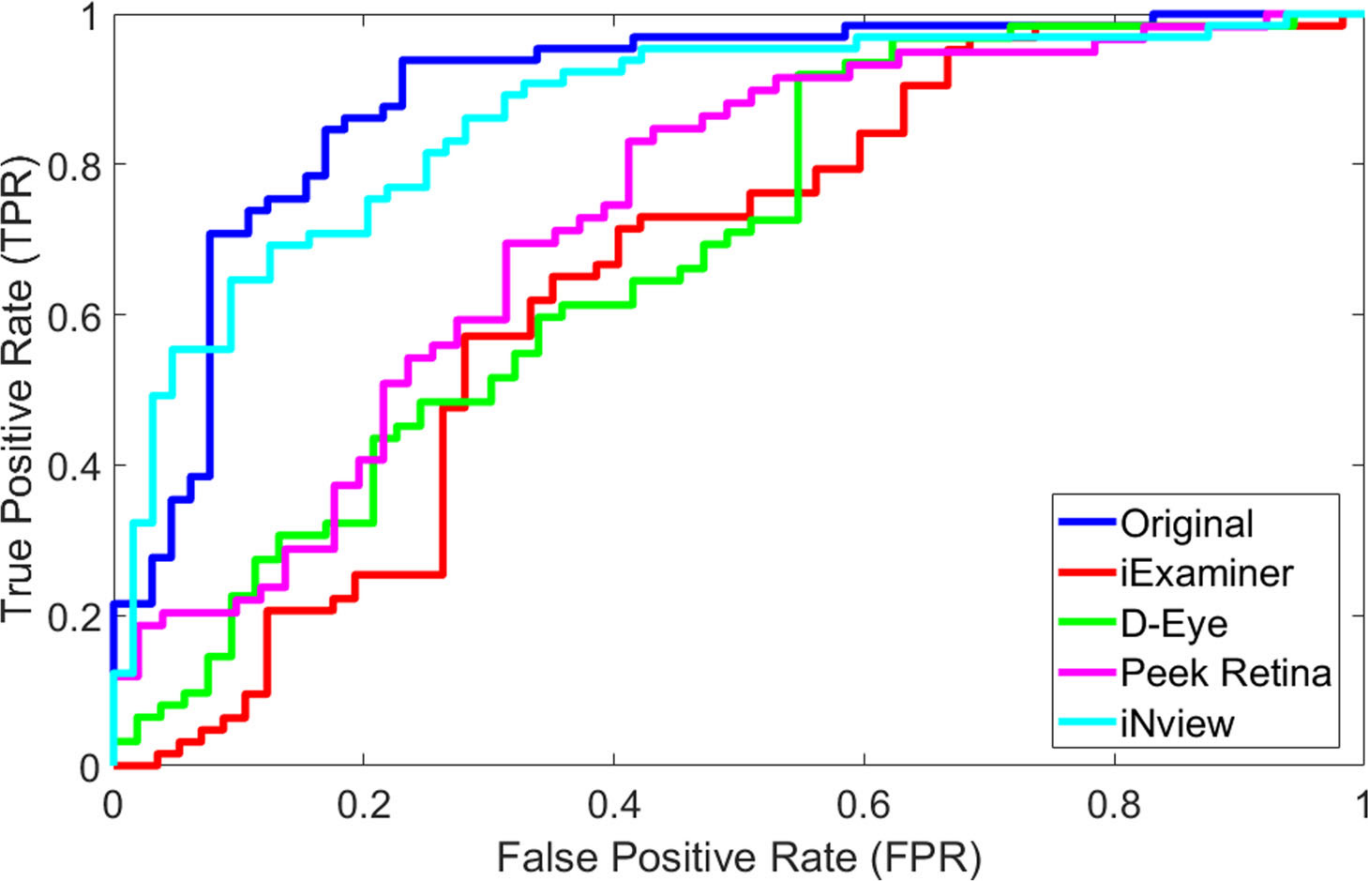
Performance analysis using ROC for different testing images from original and synthetic smartphone-based retinal images

Figure 10 compares the performance analysis of deep learning network using receiver operating characteristic (ROC) curve for different testing images from original and synthetic smartphone-based retinal images. To plot the ROC curve, we calculated the true positive rate (TPR) and the false positive rate (FPR) by changing the threshold value for the probability output of deep learning network. TPR is the probability of detecting No DR images as No DR, also known as sensitivity and recall. FPR is the probability of false alarm where No DR image is classified as DR and it is calculated as (1- specificity). ROC analysis helps to select optimal threshold value for a specific dataset discarding the suboptimal solutions due to the class distributions. As threshold value changes, the TPR and FPR change accordingly. Based on the specific system requirement, we can select any threshold value to lower the false alarm rate or increase the detection accuracy.

A specific threshold value in ROC generates an equal error value for false positive and false negative rate that is also known as equal error rate (EER). For lower EER, the overall accuracy is higher. For original, iExaminer, D-Eye, Peek Retina, and iNview datasets respectively, EERs are calculated as 16.92%, 35.09%, 39.52%, 31.37%, and 23.44% for the following threshold values, 0.6283, 0.3915, 0.3007, 0.2625, and 0.7029. The area under the curve (AUC) values for the ROC curves are 0.8835, 0.6536, 0.6826, 0.7318, and 0.8649 for original, iExaminer, D-Eye, Peek Retina, and iNview datasets, respectively as shown in Table 3. As can be seen in ROC curves, original retina images marked with solid blue line presents the best result compared with other smartphone-based retinal image datasets where all retinal structures are included in the input images, including the optic nerve, fovea, macula, and blood vessels. Since the network is trained with retina images that have a large field of view, where almost 100 degree of the retina is visible. However, smartphone-based retinal systems can visualize a smaller area from the retina. iNview shows the best among others and close the results with original images because iNview covers almost the same amount of area (94%) compared to the fundus camera. When the smaller area is captured from the retina, the network performance is the lower as shown in Fig. 10. We observed that the performance of the network depends on the field of view of the retinal imaging system.

**Table 3:** Area under the curve (AUC), Equal Error Rate (EER), and Selected Threshold for EER parameters at ROC curve for different testing images from original and synthetic smartphone-based retinal images

	AUC	Equal Error Rate	Threshold for ERR
Original	0.8835	0.1692	0.6283
iExaminer	0.6536	0.3509	0.3915
D-Eye	0.6826	0.3962	0.3007
Peek Retina	0.7318	0.3137	0.2625
iNview	0.8649	0.2344	0.7029

## Conclusions

This paper first investigated the smartphone-based portable retinal imaging systems, namely iExaminer, D-Eye, Peek Retina, and iNview to compare their image quality. Then, we adapted a deep learning framework using transfer learning. Using smartphones is an emerging research area in designing small-sized, low-power, and affordable retinal imaging systems to perform DR screening and automated DR detection due to the size, weight, and price of fundus cameras. Smartphone-based portable retinal imaging systems available on the market are capable of capturing and saving retina images without analyzing them using any image processing techniques to detect diabetic retinopathy development. Based on the results, iNview retinal imaging system has the largest field of view and better image quality compared with iExaminer, D-Eye, and Peek Retina systems. The overall classification accuracies of smartphone-based systems are sorted as 61%, 62%, 69%, and 75% for iExaminer, D-Eye, Peek Retina, and iNview images, respectively. We observed that the network DR detection performance decreases as the field of view of the smartphone-based retinal systems get smaller, where iNview is the largest and iExaminer is the smallest. The smartphone-based retina imaging systems can be used as an alternative to the direct ophthalmoscope. However, the field of view of the smartphone-based retina imaging systems plays an important role in determining the automatic DR detection accuracy.

## Data Availability

The datasets generated and analyzed during the current study are available in the Kaggle repository, https://www.kaggle.com/c/diabetic-retinopathy-detection.

## Abbreviations

DR: Diabetic retinopathy
FDA: Food and drug administration
ARIA: Automatic retinal image analysis
CNN: Convolutional neural network
FOP: Fundus on phone
AoV: Angles of retinal views
ReLU: Rectified linear units
SGD: Stochastic gradient descent
ROC: Receiver operating characteristic
TPR: True positive rate
FPR: False positive rate
AUC: Area under the curve
